# 

**Published:** 1908-03-14

**Authors:** 


### DETENTION UNDER THE LUNACY ACTS.

A few weeks ago we published in the section of The Hospital devoted to mental diseases the complaint of a correspondent that her mother was improperly detained in an asylum, and gave our reasons for concluding that the complaint was unfounded. Last week the matter came before a divisional Court on an application taken out by our correspondent for a rule for a writ of habeas corpus. The Divisional Court came to the same conclusion as that expressed in these columns, and refused the application.

### "THE CAUSE OF THE CHILDREN."

As was to be expected, the Children's Bill recently introduced into Parliament has the cordial support of the National Society for the Prevention of Cruelty to Children, which has long laboured in the cause of suffering childhood. The extent and magnitude of the society's work in safeguarding the lives of children throughout the land and rescuing them from systematic cruelty, starvation, and neglect is seldom realised. The records for the past month show that no less than 3,888 cases were dealt with, affecting the lives of 11,426 children. It is sad to note that 86 of these cases ended in death. It is also a significant fact that 3,022 children were known to be insured for a total of £15,607. The aim of the society is "that every child in the land shall live an endurable life." Great as its efforts have been in this direction there is room for considerable extension. There are districts yet untouched where children suffer and where, if funds permitted, inspectors would be placed without delay.

### BOOKS RECEIVED.

Cassell and Company.

"The Romance of Medicine." By R. C. Macfie, M.A., M.B.

Methuen and Company.

"Functional Nerve Diseases." By A. T. Schofield, M.D.

Henry Frowde and Hodder and Stoughton.

(Oxford Medical Publications.)

"Rotunda Practical Midwifery." By E. Hastings Tweedy, M.D., and G. T. Wrench, M.D.

"Rotunda Midwifery for Nurses and Midwives." By G. T. Wrench, M.D.

"A System of Medicine," Vol. III. By W. Osler, M.D., and T. McCrae, M.D.

J. Nisbet and Company, Limited.

"Nisbet's Medical Directory, 1908."

The Clarendon Press, Oxford.

"Medicine in the British Isles." By Norman Moore, M.D.

University Tutorial Press.

"School Hygiene." By Robt. A. Lyster, M.B.

### THE GRADUATES' MEDICAL SCHOOLS.

### Diary for the week March 16 to 21.

**THE ROYAL COLLEGE OF SURGEONS.**

**At 5 p.m.** Arris and Gale Lectures.
**March 16**, Dr. F. A. Bainbridge, **The Pathology of Acid Intoxication.**
**March 18 and 20**, Mr. M. Greenwood, jun., **The Physiological and Pathological Effects which follow Exposure to Compressed Air.**

**ROYAL COLLEGE OF PHYSICIANS, Pall Mall East.**

**At 5 p.m.** Gulstonian Lectures.
**March 17 and 19**, Dr. Herbert French, **The Influence of Pregnancy on Certain Medical Diseases, and of Certain Medical Diseases on Pregnancy.**

**MEDICAL GRADUATES' COLLEGE AND POLYCLINIC, Chenies Street, W.C.**

**At 5.15 p.m.**
**March 16**, Dr. F. M. Sandwith, **Bilharziasis.**
**March 17**, Mr. F. Eve, **Scme Surgical Considerations.**
**March 18**, Dr. J. H. Thursfield, **Post-basal Meningitis.**
**March 19**, Dr. L. Guthrie, **Precocious Development.**

**THE HOSPITAL FOR SICK CHILDREN, Great Ormond Street, W.C.**

**At 4 p.m.**
**March 19**, Dr. Poynton, **Rheumatism in Children under Five Years.**

**NORTH-EAST LONDON POST-GRADUATE COLLEGE, Prince of Wales's General Hospital, Tottenham, N.**

**At 4.30 p.m.**
**March 19**, Mr. Howell Evans, **Congenital Dislocation of the Hip Joint.**

**CENTRAL LONDON THROAT and EAR HOSPITAL Gray's Inn Road, W.C.**

**At 4 p.m.**
**March 19**, Dr. Atkinson, **Syphilis of the Throat.**

**LONDON SCHOOL OF CLINICAL MEDICINE, Seamen's Hospital, Greenwich.**

**At 2.15 p.m.**
**March 19**, Dr. Rankin, **Chorea.**

**THE POST-GRADUATE COLLEGE, West London Hospital, Hammersmith, W.**

**At noon.**
**March 16**, Dr. Low, **Pathological Demonstration.**
**March 17 and 19**, Dr. Pritchard, **Practical Medicine.**
**At 5 p.m.**
**March 16**, Dr. Bull, **Diseases of the Tonsils—I.**
**March 17**, Dr. Bull, **Diseases of the Tonsils—II.**
**March 18**, Dr. Beddard, **Practical Medicine—V.**
**March 19**, Mr. Baldwin, **Practical Surgery—VI.**
**March 20**, Dr. Hood, **Arteriosclerosis.**

**HOSPITAL FOR CONSUMPTION AND DISEASES OF THE CHEST, Brompton, S.W.**

**At 4 p.m.**
**March 18**, Dr. Grey, **Demonstration of Cases from the Throat Department.**

**NATIONAL HOSPITAL FOR THE PARALYSED AND EPILEPTIC, Queen Square, Bloomsbury, W.C.**

**At 3.30 p.m.**
**March 17**, Dr. Collier, **Torticollis and the Tics.**
**March 20**, Dr. F. Buzzard, **Acute Infection of the Brain and Membranes.**

